# Knockdown of ANP32E inhibits colorectal cancer cell growth and glycolysis by regulating the AKT/mTOR pathway

**DOI:** 10.1515/biol-2022-0817

**Published:** 2024-03-09

**Authors:** Jiaojiao Liu, Yanchao Liu, Qi Zhao

**Affiliations:** Department of Clinical Laboratory, Beihua University Affiliated Hospital, No. 12, Jiefang Middle Road, Jilin, Jilin, 132011, China; Department of Clinical Laboratory, Jilin Gynecology and Obstetrics Hospital, Jilin, Jilin, 130211, China

**Keywords:** ANP32E, glycolysis, CRC, AKT/mTOR pathway

## Abstract

Colorectal cancer (CRC) is the third most common tumor, with an increasing number of deaths worldwide each year. Tremendous advances in the diagnosis and treatment of CRC have significantly improved the outcomes for CRC patients. Additionally, accumulating evidence has hinted the relationship between acidic nuclear phosphoprotein 32 family member E (ANP32E) and cancer progression. But the role of ANP32E in CRC remains unclear. In our study, through TCGA database, it was demonstrated that the expression of ANP32E was enhanced in COAD tissues (*n* = 286). In addition, the mRNA and protein expression of ANP32E was also confirmed to be upregulated in CRC cell lines. Further investigation uncovered that knockdown of ANP32E suppressed cell proliferation and glycolysis, and facilitated cell apoptosis in CRC. Moreover, inhibition of ANP32E inhibited the AKT/mTOR pathway. Through rescue assays, we discovered that the reduced cell proliferation, glycolysis and the enhanced cell apoptosis mediated by ANP32E repression was reversed by SC79 treatment. In summary, ANP32E aggravated the growth and glycolysis of CRC cells by stimulating the AKT/mTOR pathway. This finding suggested that the ANP32E has the potential to be explored as a novel biomarker for CRC treatment.

## 1 Introduction

Colorectal cancer (CRC) is the third most prevalent cancer type on earth [1,2,3]. Most CRC patients are not diagnosed until late stage [4]. Unfortunately, there are only limited effective treatment options available for advanced CRC patients [5,6]. Many techniques and methods for early diagnosis exist and are still used today, currently, targeted therapy is more and more favored by researchers [7,8]. Therefore, it is imperative to understand the molecular mechanism of CRC progress and identify precise and useful biomarkers.

Increasing proteins as biomarkers have been shown to modulate cancer progression, including CRC. For example, glutathione *S*-transferase pi 1 enhances STAT3 in CRC to facilitate tumor growth and metastasis [9]. The RNA binding protein neuro oncological ventral antigen 1 (NOVA1) induces the JAK2-STAT3 signaling pathway through modulating IL-6 mRNA stability in CRC [10]. Additionally, spondin 2 activates PYK2 to accelerate M2-polarized tumor-associated macrophage infiltration and CRC progression [11]. Moreover, monocarboxylate transporter 1 enhances autophagy to retard osimertinib-stimulated CRC suppression through modulating the LKB1/AMPK signaling [12].

Acidic nuclear phosphoprotein 32 family member E (ANP32E) belongs to the leucine rich protein family, which is involved in multiple biological processes, such as cell adhesion, early mammalian cell growth, and cancer metastasis [13,14]. ANP32E has been revealed to participate in the progression of different cancers. For instance, ANP32E is related with the poor prognosis of pancreatic cancer and contributes to cell proliferation and migration [15]. In addition, lncRNA NORAD targets miR-202-5p/ANP32E axis in pancreatic cancer to exacerbate stemness and self-renewal [16]. Furthermore, ANP32E upregulates E2F1 in triple-negative breast to stimulate tumorigenesis [17]. ANP32E enhances glycolysis in thyroid carcinoma to aggravate cell proliferation and migration [18]. Moreover, hsa-let-7c suppresses ANP32E in lung adenocarcinoma to retard tumorigenesis [19]. Importantly, TCGA database has demonstrated that ANP32E is highly expressed in COAD, but its regulatory roles and the correlative mechanism in CRC progression remain unclear. Therefore, more investigations are needed to uncover its role and function in CRC.

The aim of this study is to investigate the regulatory functions of ANP32E and related pathway in CRC progression. In our study, results demonstrated that ANP32E aggravated CRC progression by stimulating the AKT/mTOR pathway, suggesting that ANP32E might be a potential therapeutic target for CRC treatment.

## 2 Materials and methods

### 2.1 Cell lines and cell culture

The CRC cell lines (SW480, HCT116, SW620, and SW837) and the normal colonic cell line NCM460 were acquired from the American Type Culture Collection (ATCC, Manassas, VA, USA). Dulbecco’s modified Eagle’s (DMEM, Sigma, St Louis, MO, USA) medium containing 10% fetal bovine serum (FBS, Hyclone, Logan City, UT, USA) was utilized to culture these cells at 37°C in a moist incubator with 5% CO_2_. SC79 (AKT stimulator) was employed to treat CRC cells.

### 2.2 Transfection

The small interfering RNA (siRNA) designed to knockdown ANP32E (si-ANP32E#1 and si-ANP32E#2), and its negative control (si-NC) were synthesized by GenePharma (Shanghai, China). Transfection of the above siRNAs (50 nM, 48 h) into HCT116 and SW480 cells were conducted through Lipofectamine 3000 reagent (Invitrogen).

### 2.3 RT-qPCR

The RNAs extracted from CRC cells with the TRIzol reagents (Invitrogen, Carlsbad, CA) were reversely transcribed into cDNAs through the PrimeScript™ RT Master Mix kit (Takara, Dalian, China). The SYBR Premix Ex Taq^™^ (Takara, Shanghai, China) was applied for qRT-PCR. PCR was performed: 95°C for 10 min, 55°C for 2 min, 72°C for 2 min, followed by 40 cycles of 95°C for 15 s, and 60°C for 1 min. The data quantification was done through the 2^−ΔΔCt^ method, normalizing to GAPDH.

The primer sequences were as followed:

ANP32E forward, 5′-TGCCTGTGTGTCAATGGGG-3′, and reverse, 5′-GCAGAGCTTCTACTGTACTGAGA-3′;

GAPDH forward, 5′-TAACTCTGGTAAAGTGGATATTG-3′, and reverse, 5′-GAAGATGGTGATGGGATTTC-3′.

### 2.4 Western blot

The proteins extracted with RIPA lysis buffer (Thermo Fisher Scientific, Inc.) were segregated through 10% SDS-PAGE and transferred onto PVDF membranes (Beyotime, Shanghai, China). After blocking with skim milk, the membranes were incubated with primary antibodies at 4°C for 12 h. Next the appropriate HRP-conjugated secondary antibodies (1:2,000; ab7090; Abcam, Shanghai, China) were added into the membranes for incubation. GAPDH acted as the internal reference. Finally, the chemiluminescence detection kit (Thermo Fisher Scientific, Inc.) was employed to evaluate the bands [20].

The primary antibodies included anti-ANP32E (1 µg/mL; ab5993; Abcam), GLUT1 (1/100,000; ab115730), HK2 (1:1,000; ab104836), Bax (1:1,000; ab32503), Bcl-2 (1:2,000; ab182858), p-AKT (1:1,000; ab38449), AKT (1:500; ab8805), p-mTOR (1:1,000; ab109268), mTOR (1:1,000; ab32028), and β-actin (1 µg/mL; ab8226) antibodies.

### 2.5 CCK-8 assay

CRC cells were plated in 96-well plate and cultured for 0, 24, 48, and 72 h. Then, 10 μL of CCK-8 reagent (Sigma-Aldrich, St. Louis, MO, USA) was added into cells and incubated for 4 h. The microplate reader (SpectraMax M5, Molecular Devices, San Jose, CA) was adopted to examine the OD value at 450 nm.

### 2.6 Colony formation assay

First, CRC cells were seeded onto the 6-well plates for incubation at 37°C. After 14 days, cells were fixed with 4% paraformaldehyde and stained with 1% crystal violet dye for 15 min. Finally, the microscope (Olympus, Japan) was adopted to capture images and then count the colonies.

### 2.7 Detection of ATP level, glucose consumption, and lactate production

48 h after transfection, CRC cells and the culture medium were collected. Glycolysis was estimated according to ATP level in CRC cells, glucose consumption in culture medium, and lactate production in culture medium through the ATP Assay Kit (Abcam, CA, USA), Glucose Uptake Assay Kit (Abcam), and Lactate Assay Kit-WST (Abcam), respectively.

### 2.8 Flow cytometry

The Annexin V-FITC/PI apoptosis kit (Nanjing KeyGen Biotech Co., Ltd.) was utilized to evaluate cell apoptosis. After being harvested and resuspended, the CRC cells were mixed with the buffer involving 5 µL Annexin V-fluorescein isothiocyanate (Annexin V-FITC) and 5 µL propidium iodide (PI). The cell apoptosis was analyzed through the flow cytometer (Thermo Fisher Scientific, Rockford, IL, USA) [21]. Q1: Mechanical injury necrosis; Q2: Late apoptosis; Q3: Early apoptosis; Q4: Normal cells. Forward scatter, side scatter: Displays cell location and size. Cross door diagram: FL1: FTIC; FL2: PI.

### 2.9 Statistical analysis

The data were shown as mean value ± standard deviation. SPSS 20.0 software (IBM, USA) and GraphPad Prism 8.0 software were adopted for statistical analysis. ImageJ was utilized for detecting the intensity of protein band. All experiments were repeated in triplicate. The comparisons among groups were estimated through the Student’s *t*-test (two groups) or one-way ANOVA (multiple groups) followed by Tukey’s “post-hoc” test. *p* < 0.05 was regarded to be statistically significant.

## 3 Results

### 3.1 ANP32E expression was enhanced in CRC

**Figure 1:**
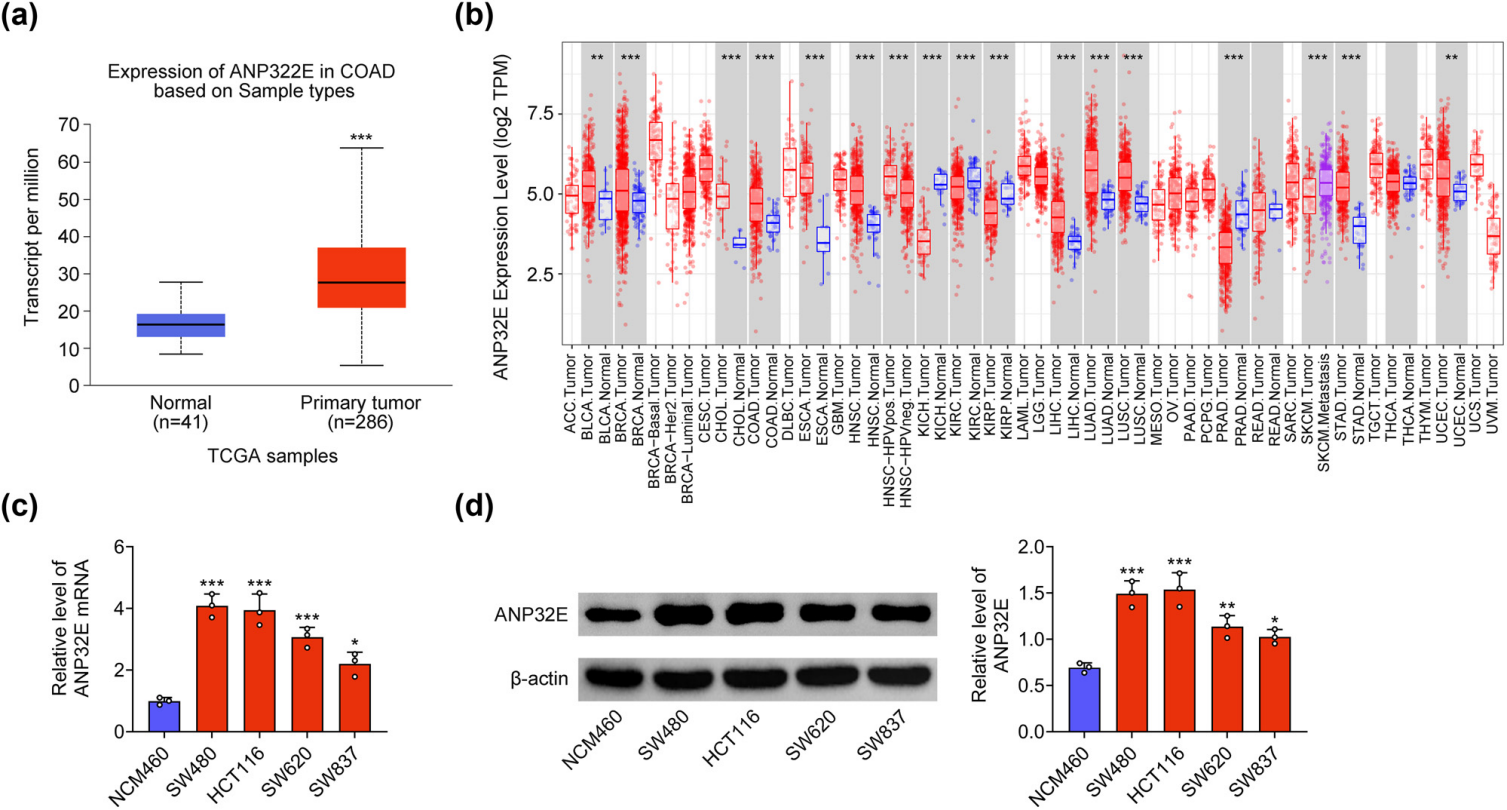
The ANP32E expression was enhanced in CRC. (a) The expression of ANP32E in COAD tissues (*n* = 286) and normal tissues (*n* = 41) was identified through TCGA database. (b) The expression of ANP32E in various cancers (normal tissues and tumor tissues) was displayed through TCGA database. (c) The mRNA expression of ANP32E was examined in CRC cell lines (SW480, HCT116, SW620, and SW837) through RT-qPCR. *N* = 3. (d) The protein expression of ANP32E was tested in CRC cell lines (SW480, HCT116, SW620, and SW837) through western blot. *N* = 3. *p < 0.05, **p < 0.01, ***p < 0.001.

First, the enhanced expression of ANP32E in COAD tissues was found through TCGA database (*n* = 286) (Figure 1a). Moreover, the expression of ANP32E was confirmed in various cancers, and it was discovered that ANP32E exhibited higher expression in COAD tissues than that in the normal tissues (Figure 1b). Next the mRNA expression of ANP32E was upregulated in CRC cell lines but not in the normal colonic cell line NCM460 cells (Figure 1c). Moreover, the similar change in the ANP32E protein expression was displayed in Figure 1d. In general, the ANP32E expression was enhanced in CRC.

### 3.2 Knockdown of ANP32E retarded cell proliferation in CRC

**Figure 2:**
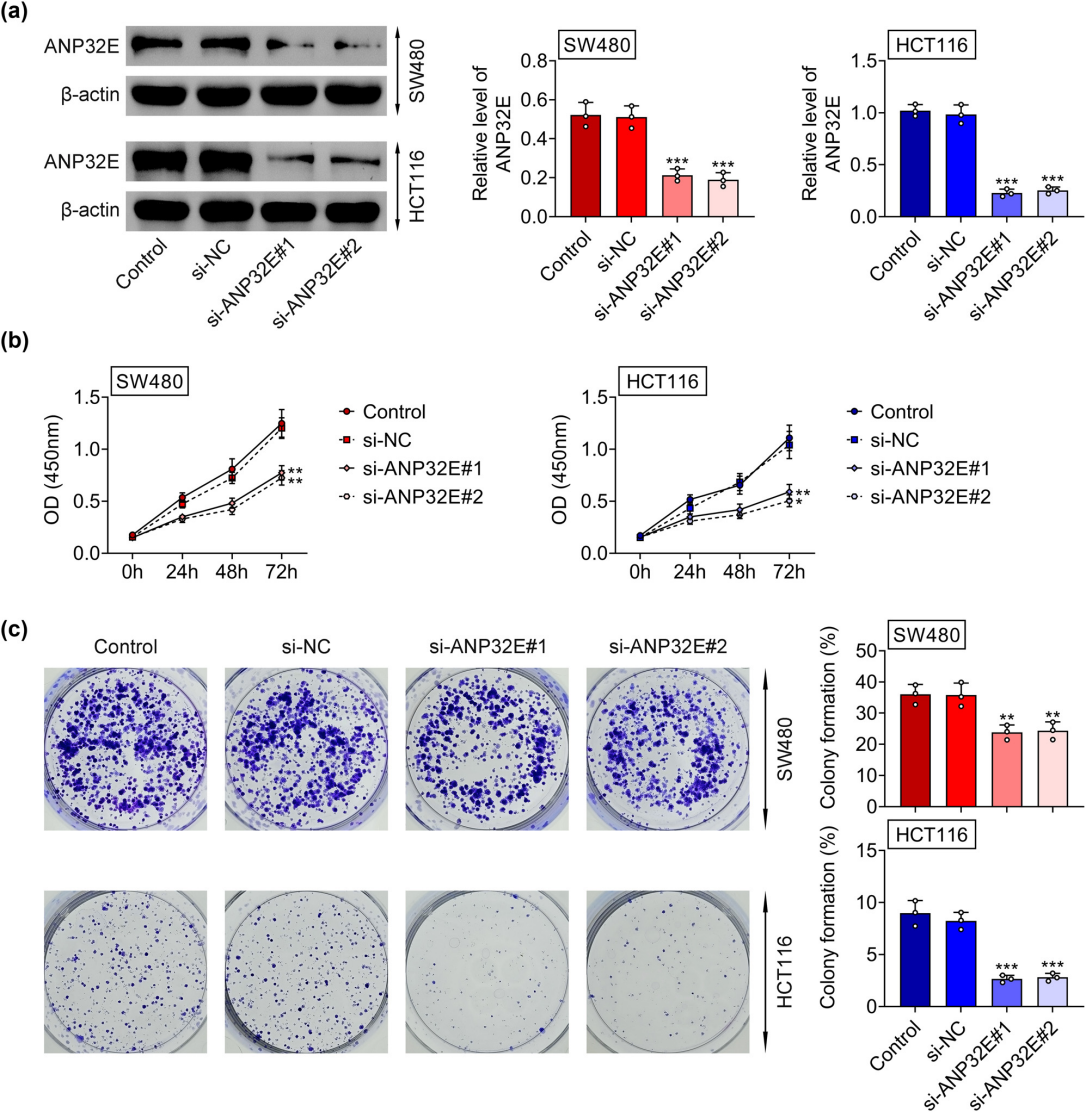
Knockdown of ANP32E retarded cell proliferation in CRC. Groups were divided into the Control, si-NC, si-ANP32E#1, and si-ANP32E#2 group. (a) The knockdown efficiency of ANP32E was confirmed in SW480 and HCT116 cells through western blot. (b and c) The cell proliferation was assessed through CCK-8 and colony formation assays. *p < 0.05, **p < 0.01, ***p < 0.001.

The knockdown efficiency of ANP32E is shown in Figure 2a. Through CCK-8 and colony formation assays, it was uncovered that the cell proliferation was reduced after ANP32E knockdown (Figure 2b and c).

### 3.3 Silencing of ANP32E weakened the glycolysis in CRC

**Figure 3:**
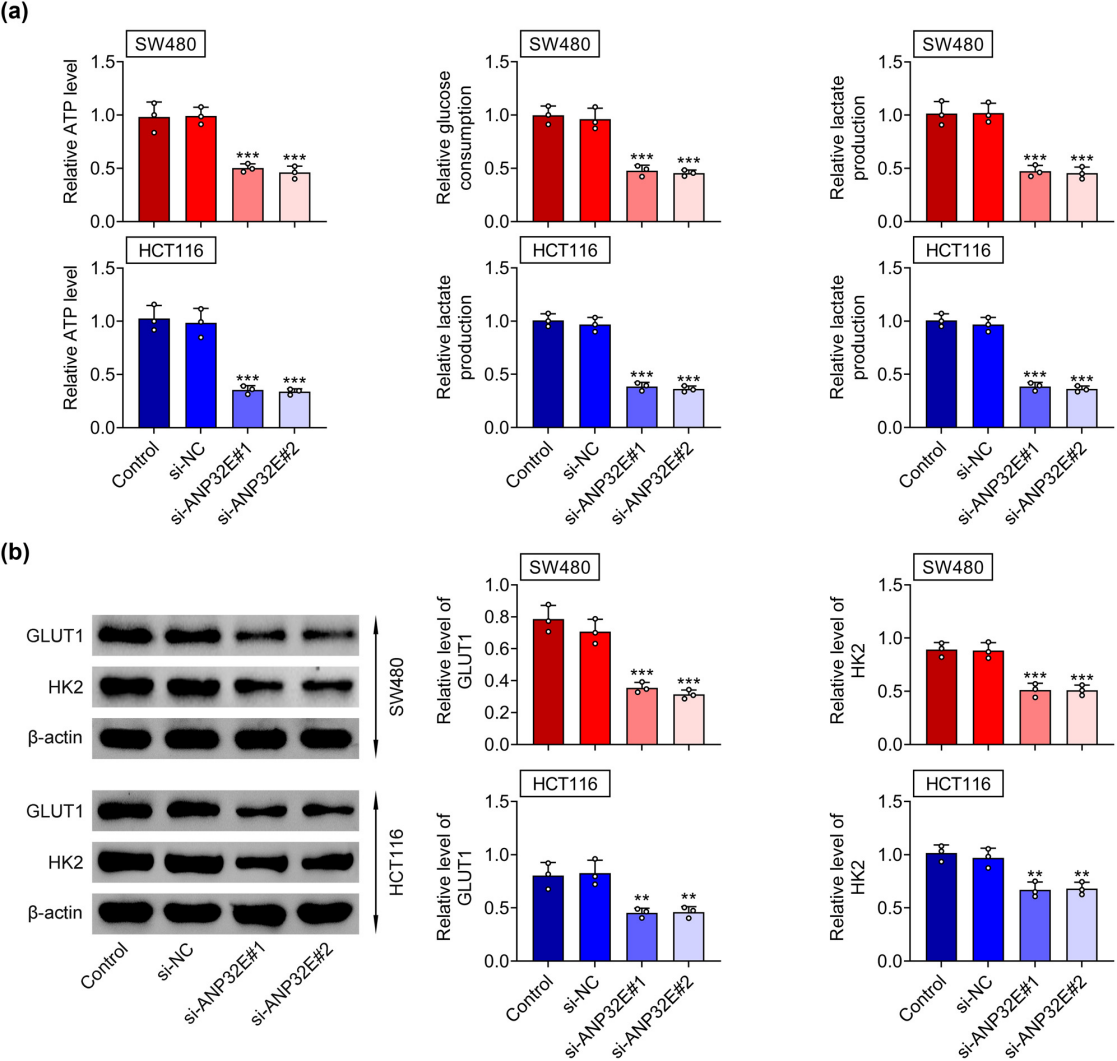
Silencing of ANP32E weakened the glycolysis in CRC. Groups were divided into the Control, si-NC, si-ANP32E#1, and si-ANP32E#2 group. (a) The ATP level, glucose consumption, and lactate production were evaluated through corresponding commercial kits. (b) The protein expression of GLUT1 and HK2 was examined through western blot. **p < 0.01, ***p < 0.001.

Further investigation focused on the glycolysis in CRC. We discovered that the ATP level, glucose consumption and lactate production were all decreased after silencing ANP32E (Figure 3a). Moreover, the levels of glycolysis related proteins (GLUT1 and HK2) were downregulated after ANP32E suppression (Figure 3b). These data indicated that silencing of ANP32E weakened the glycolysis in CRC.

### 3.4 Suppression of ANP32E facilitated cell apoptosis in CRC

**Figure 4:**
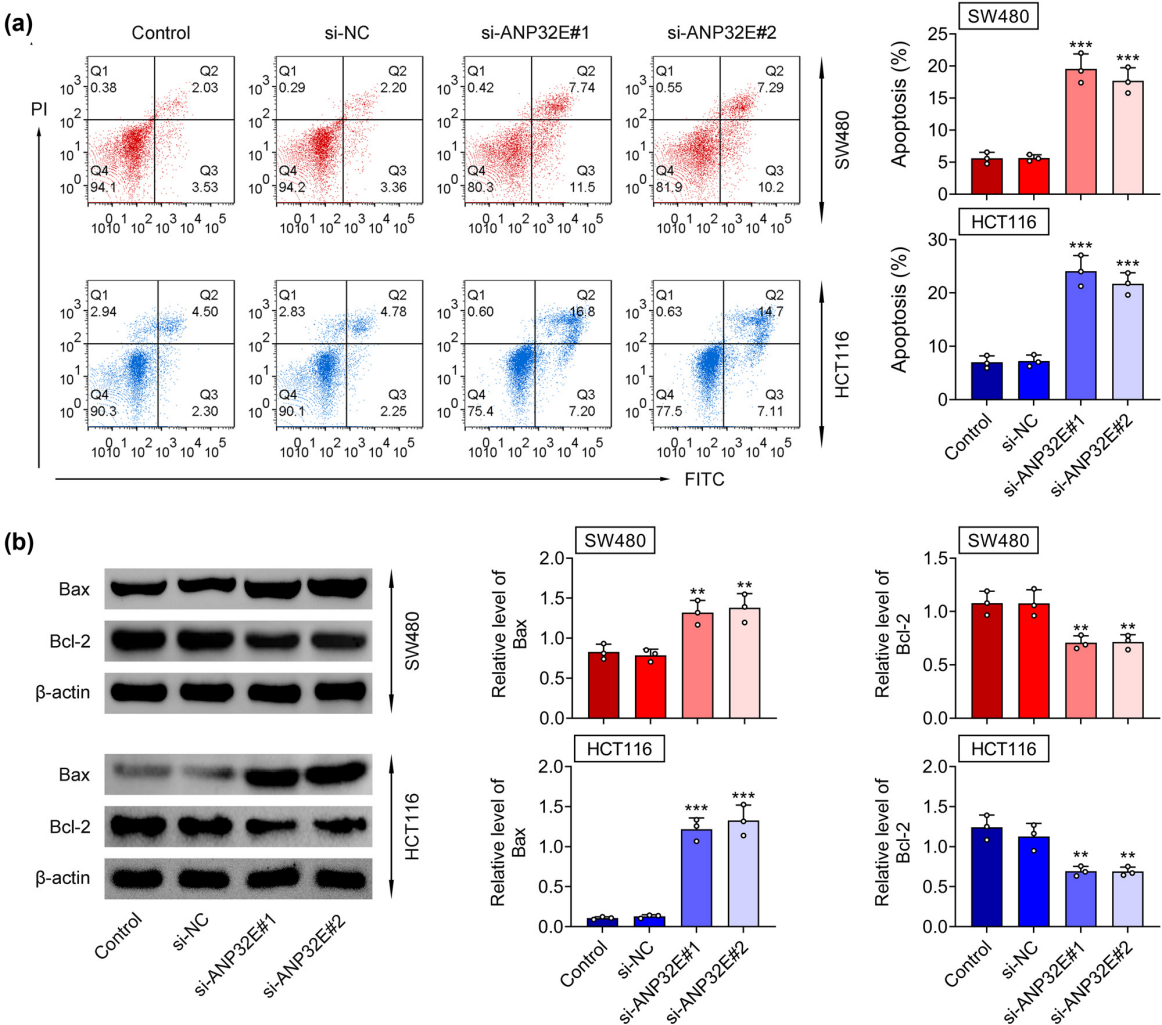
Suppression of ANP32E facilitated cell apoptosis in CRC. Groups were divided into the Control, si-NC, si-ANP32E#1, and si-ANP32E#2 group. (a) The cell apoptosis was tested through flow cytometry. (b) The protein expression of Bax and Bcl-2 was measured through western blot. **p < 0.01, ***p < 0.001.

As shown in Figure 4a, the cell apoptosis was enhanced after ANP32E knockdown. In addition, the Bax expression was enhanced, and the Bcl-2 expression was reduced after repressing ANP32E (Figure 4b). In summary, suppression of ANP32E facilitated cell apoptosis in CRC.

### 3.5 SC79 (AKT stimulator) treatment rescued the suppressed CRC progression mediated by ANP32E knockdown

**Figure 5:**
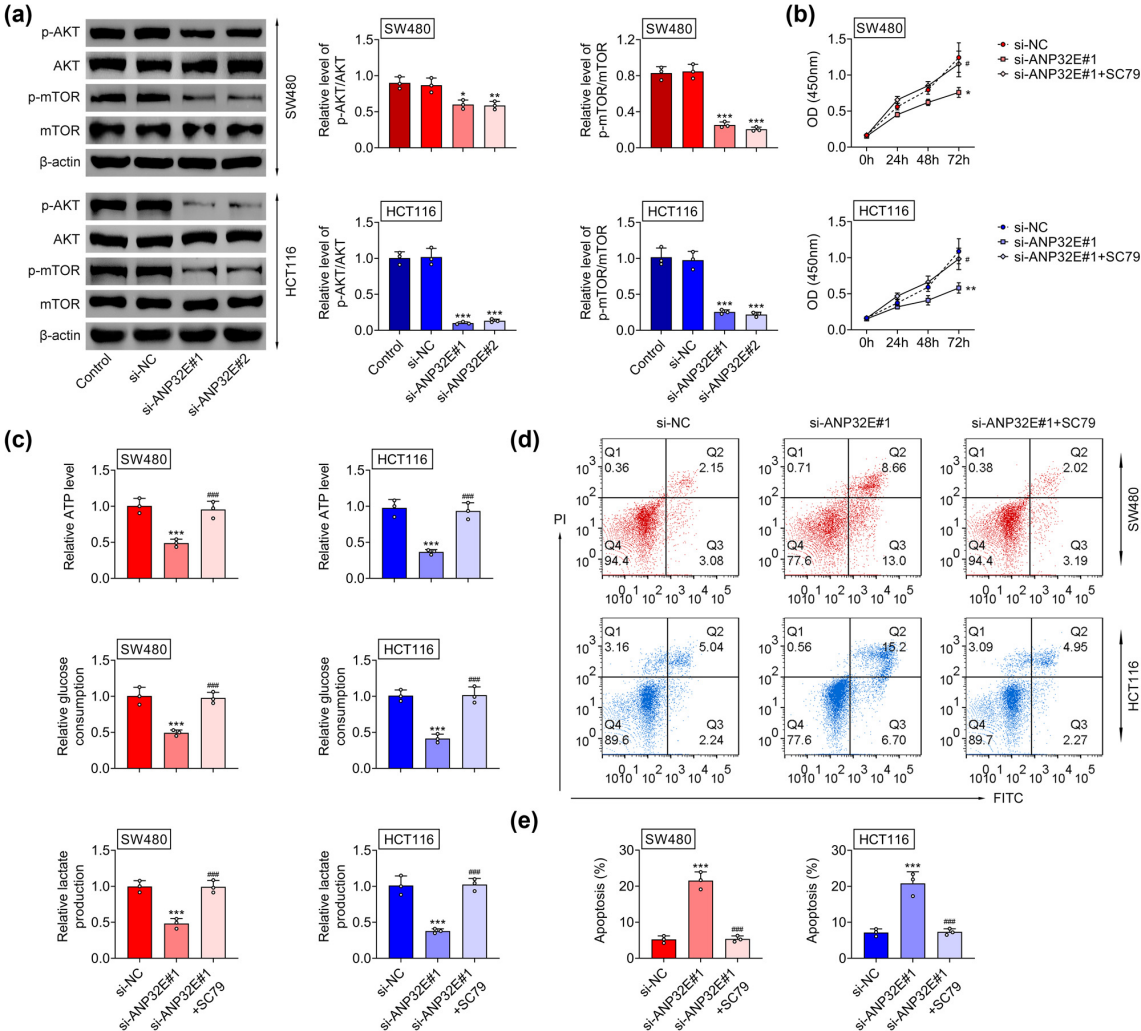
Inhibition of ANP32E affected CRC progression through modulating AKT/mTOR pathway. (a) The protein expression of AKT/mTOR pathway associated proteins (p-AKT, AKT, P-mTOR, and mTOR) was examined in the Control, si-NC, si-ANP32E#1, and si-ANP32E#2 groups through western blot. (b) The cell proliferation was detected in the si-NC, si-ANP32E#1, and si-ANP32E#1 + SC79 groups through CCK-8 assay. (c) The ATP level, glucose consumption, and lactate production were measured in the si-NC, si-ANP32E#1, and si-ANP32E#1 + SC79 groups through corresponding commercial kits. (d and e) The cell apoptosis was tested in the si-NC, si-ANP32E#1, and si-ANP32E#1 + SC79 groups through flow cytometry. *p < 0.05, **p < 0.01, ***p < 0.001 vs the si-NC group; ^#^ *p* < 0.05, ^###^ *p* < 0.001 vs the si-ANP32E#1 group.

Finally, the relationship between ANP32E and the AKT/mTOR pathway in CRC was investigated. The *p*-AKT/AKT and p-mTOR/mTOR levels were decreased after ANP32E inhibition (Figure 5a). Next the rescue assays were carried out. The cell proliferation was weakened after ANP32E suppression, but this effect could be restored by SC79 (AKT stimulator) treatment (Figure 5b). Additionally, the ATP level, glucose consumption, and lactate production were reduced after ANP32E inhibition, which were counteracted by SC79 treatment (Figure 5c). Furthermore, the elevated cell apoptosis mediated by ANP32E repression was offset by SC79 treatment (Figure 5d and e). These results revealed that ANP32E affected CRC progression through modulating AKT/mTOR pathway.

## 4 Discussion

Through TCGA database, it was demonstrated that the expression of ANP32E was enhanced in COAD tissues. In addition, the mRNA and protein expression of ANP32E was also upregulated in CRC cell lines. Further investigation uncovered that knockdown of ANP32E suppressed cell proliferation and glycolysis, and facilitated cell apoptosis in CRC. Moreover, inhibition of ANP32E retarded the AKT/mTOR pathway. Through rescue assays, it was discovered that the reduced cell proliferation, glycolysis, and the enhanced cell apoptosis mediated by ANP32E repression was reversed by SC79 (AKT stimulator) treatment.

A great number of proteins have been acknowledged to be latent oncogenic or anticancer factors in CRC [9,10,11,12]. ANP32E has been discovered to participate in a variety of cancers [15,16,17,18,19]. However, the effects of ANP32E on CRC progression remain unclear. In this study, TCGA database manifested that ANP32E is highly expressed in COAD. In addition, the mRNA and protein expression of ANP32E were also upregulated in CRC cell lines.

Metabolic abnormality is considered as a marker of cancer cells, which is a critical research field and has acquired great interest recently [22,23]. Unlike normal cells, which derive most of their energy from oxidative phosphorylation of mitochondria, cancer cells rely on aerobic glycolysis as their main energy resource, in addition to the cancer cells, bacteria (maybe as some normal cells) can be involved in aerobic glycolysis process [24,25,26]. This glycolysis has been shown to participate in CRC progression. For instance, miR-103a-3p regulates hippo/YAP1/HIF1A axis in CRC to enhance glycolysis [27]. Moreover, histone demethylase JMJD2D aggravates glycolysis and CRC progression through the HIF1 signaling pathway [28]. In addition, the deubiquitinase OTUB2 enhances glycolysis to aggravate the progression of CRC [27,29]. Therefore, further investigations are needed to clarify the effects of ANP32E on glycolysis in CRC. In our study, we uncovered that knockdown of ANP32E weakened cell proliferation and glycolysis, and facilitated cell apoptosis in CRC.

The AKT/mTOR pathway is an important modulator, that has been elucidated to be related with glycolysis. This relationship between glycolysis and Akt/mTOR pathway in CRC progression has also been investigated. For example, KLK10 modulates tumor growth and glucose metabolism in CRC through the PI3K/Akt/mTOR pathway [30]. Additionally, downregulation of FOXO6 retards the Akt/mTOR pathway in CRC to inhibit the cell proliferation and glycolysis [31]. Importantly, ANP32E stimulates the AKT/mTOR/HK2 signaling pathway to accelerate cell proliferation, migration and glycolysis in thyroid carcinoma [18]. However, the regulatory effects of ANP32E on the relationship between AKT/mTOR pathway and glycolysis in CRC progression need more investigations. In this study, inhibition of ANP32E retarded the AKT/mTOR pathway. Through rescue assays, we discovered that the reduced cell proliferation, glycolysis, and the enhanced cell apoptosis mediated by ANP32E repression was reversed by SC79 treatment.

This study for the first time investigated the regulatory function of ANP32E on tumor growth and glycolysis in CRC. Our findings revealed that ANP32E aggravated CRC tumor growth and glycolysis through stimulating the AKT/mTOR pathway. Our findings suggested that ANP32E may be a prospective bio-target for CRC treatment. However, some limitations are exhibited in this work, such as lacking more human samples, animal models, and other biological processes (autophagy, mitochondria damage, exosomes, immune escape and so on). In the future, more experiments will be conducted to confirm the role of ANP32E in CRC.
